# Analysis of Two Large Earthquakes in the South Pacific Coast of Mexico Using Rényi Entropy

**DOI:** 10.3390/e28101159

**Published:** 2026-10-21

**Authors:** Carlos Carrizales-Velazquez, Israel Reyes-Ramírez, Fernando Angulo-Brown, Lev Guzmán-Vargas

**Affiliations:** 1 Laboratorio de Sistemas Complejos, Unidad Interdisciplinaria en Ingeniería y Tecnologías Avanzadas, Instituto Politécnico Nacional, Ciudad de México 07340, Mexico; ireyesr@ipn.mx; 2 Unidad Profesional Interdisciplinaria en Ingeniería Campus Tlaxcala, Instituto Politécnico Nacional, Tlaxcala de Xicohténcatl 90000, Mexico; 3 Departamento de Física, Escuela Superior de Física y Matemáticas, Instituto Politécnico Nacional, Ciudad de México 07738, Mexico; fangulob@ipn.mx

**Keywords:** Rényi entropy, large-magnitude events, critical transitions

## Abstract

We present a study on the behavior of Rényi entropy for earthquake magnitudes, focusing on two large-magnitude events in the Pacific coast of Mexico: the M8.2 Tehuantepec earthquake in 2017 and the M7.7 Michoacán earthquake in 2022. Based on the discrete version of Rényi entropy, we show that entropy decreases before the mainshocks occur and subsequently exhibits an abrupt increase before returning to stable values. The behavior of Rényi entropy in terms of the control parameter α, which either rewards or penalizes regions of the distribution, exhibits differences in the rate at which entropy decreases as α increases before, during, and after an earthquake. This abrupt change in entropy exhibits characteristics analogous to those observed in critical transitions.

## 1. Introduction

In recent decades, seismic sequences (seismic catalogs) have been studied using methods from complex systems theory [1,2,3,4,5]. In particular, Shannon entropy [6]—derived from information theory—has been employed. As is well known, the concept of entropy was introduced by Clausius within the context of classical thermodynamics (1865) and was subsequently interpreted by Boltzmann (1872) to link it to the number of possible microstates of a thermodynamic system in equilibrium. In 1948, Shannon extended the concept of entropy through information theory. Throughout the 20th century, the concept of entropy was generalized by various authors, such as Rényi and Tsallis [7,8]. In recent decades, entropy analysis has been established as a robust mathematical framework for investigating the complex dynamics of seismic sequences in various tectonic settings. A number of studies have effectively utilized metrics from information theory and thermodynamics to identify precursor signals and comprehend the statistical mechanics of earthquake generation. For instance, Varotsos et al. [9] and Sarlis et al. [10] demonstrated that major seismic events in Japan and Mexico are preceded or accompanied by a significant increase in entropy associated with the natural time domain, indicating a critical transition in the regional stress state. In a similar vein, researchers have observed significant variations in structural predictability around major events. Specifically, Telesca et al. [11], De Santis et al. [12], Posadas et al. [13] and Posadas et al. [14] reported clear transitions in Shannon entropy following the occurrence of major earthquakes in seismically active regions of Italy, Spain and Chile. This series of studies has led to a better understanding of seismic phenomena, yielding interesting results that reveal reproducible patterns of entropy as a function of time, consistent with the seismic cycle. Although these results based on Shannon entropy reveal important features and changes in the evolution of the level of irregularity in seismic activity, these analyses have been limited to evaluating entropy without considering differentiated weights, which would allow for assigning greater or lesser importance to specific parts of a probability distribution based on a control parameter, as is the case with Rényi entropy. Another important aspect is the discretization of Shannon and Rényi entropies, which is necessary for an adequate estimation of these metrics in the case of earthquake magnitude sequences where the resolution of the catalogs is limited. In this paper, we present a study using Rényi entropy to analyze two mainshocks that occurred in the seismic zone of the southern Mexican Pacific, with magnitudes of M7.7 and M8.2, respectively. Our results show that entropy increases abruptly with the occurrence of these large-magnitude earthquakes, and exhibits variations consistent with a decrease to a minimum value prior to the large earthquake, after which it returns to stable values consistent with a dynamic quasi-equilibrium. The present article is organized as follows: in Section 2, we describe the methodology used in our analysis; in Section 3, the seismic database is presented; Section 4 reports on the application of Rényi entropy trough our database are showed; finally, the discussion and conclusions are summarized in Section 5.

## 2. Methodology

### 2.1. Estimation of the Probability Density Function of Seismic Activity

The estimation of the probability density function (PDF) of seismic activity has been the subject of investigation in a variety of studies [12,14,15,16], in which earthquake magnitude is used as the random variable that characterizes the state of the system within a given region. In the present work, we propose an alternative methodology for estimating the PDF of earthquake magnitudes. In 1944, Beno Gutenberg and Charles F. Richter introduced an empirical relationship describing the cumulative number of seismic events with a magnitude greater than or equal to M [17]. This relationship is now widely known as the Gutenberg–Richter (GR) law:

(1) $$\log_{10}N\left(M\right)=a-bM,$$

where the parameter *a* reflects the overall seismic activity within a given space–time region and the parameter *b* characterizes the relative frequency of small and large earthquakes. These parameters (*a* and *b*) depend on the seismic catalog and can be estimated using various statistical approaches, including least-squares fitting and maximum-likelihood estimation [17,18,19,20]. In practice, Equation (1) is valid only for magnitudes $M\geq M_{c}$, where $M_{c}$ denotes the magnitude of completeness, i.e., the lowest magnitude above which the seismic catalog is considered complete [19,21,22,23,24].

An important observation is that normalizing N(M) in Equation (1) yields the survivor function S(M), which is related to the cumulative distribution function F(M) by S(M)=1−F(M). Consequently, the corresponding probability density function is given by P(M)=dF(M)/dM=−dS(M)/dM. Therefore, taking $N\left(M_{c}\right)=10^{a-bM_{c}}$ as the normalization factor, the probability density function P(M) is given by

(2) $$P\left(M\right)=b\ln\left(10\right)10^{-b(M-M_{c})}.$$

On the other hand, the Aki–Utsu maximum-likelihood method assumes that earthquake magnitudes above the completeness magnitude $M_{c}$ follow an exponential distribution characterized by the lower bound $M_{c}$ and the average magnitude $\bar{M}$, of the form [18],

(3) $$P\left(M\right)=\frac{1}{\bar{M}-M_{c}}\exp\left(-\frac{M-M_{c}}{\bar{M}-M_{c}}\right).$$

By comparing Equations (2) and (3), we obtain

(4) $$\log_{10}\left(b\ln10\right)-b\left(M-M_{c}\right)=-\log_{10}\left(\bar{M}-M_{c}\right)-\frac{M-M_{c}}{\ln10(\bar{M}-M_{c})}.$$

By identifying the coefficients of *M* in Equation (4), we obtain

(5) $$b\ln\left(10\right)=\frac{1}{\bar{M}-M_{c}},$$

This result is consistent with several previous studies [14,15,16,18,25]. Moreover, Utsu [26] showed that the completeness magnitude should be corrected according to the following relationship,

(6) $$M_{c}^{u}=M_{c}-\frac{\delta_{M}}{2},$$

where $M_{c}^{u}$ denotes the Utsu-corrected completeness magnitude and $\delta_{M}$ is the smallest resolution reported in seismic catalogs (typically $\delta_{M}=0.1$). Accordingly, Equation (2) can be rewritten as

(7) $$P\left(M\right)=\frac{10^{-b(M-M_{c}^{u})}}{\bar{M}-M_{c}^{u}}.$$

We notice that Equation (7) satisfies the necessary condition of $\int_{M_{c}^{u}}^{\infty }P\left(M\right)dM=1$ [27]. It is important to note that alternative P(M) proposals in the literature fail to satisfy this property [14]. Throughout this subsection, all calculations have been carried out under the assumption that the Gutenberg–Richter law is continuous. In practice, however, the Gutenberg–Richter law is evaluated only at discrete magnitude values that are multiples of $\delta_{M}$. In the next subsection, we discuss an important mathematical consequence of this discretization.

### 2.2. Shannon Information Entropy of Earthquake Magnitudes

The Shannon entropy associated with the distribution of earthquake magnitudes is defined as [14,16]

(8) $$H\left(M\right)=-\int_{M_{c}^{u}}^{\infty }P\left(M\right)\ln P\left(M\right)dM.$$

It is important to note that Equation (8) is defined for a continuous random variable, whereas earthquake magnitudes *M* reported in seismic catalogs are recorded with a finite resolution, typically $\delta_{M}=0.1$ [14]. However, some seismic catalogs may provide a higher-magnitude resolution; for example, the USGS reports a resolution of $\delta_{M}=0.01$. Therefore, the value of $\delta_{M}$ may vary among seismic catalogs, and this variability is explicitly accounted for in the procedure described in this work. Accordingly, Equation (8) is reformulated to account for the finite resolution of the reported magnitude data. The relationship between differential entropy and discrete entropy is developed in detail in [28]. In particular, it is shown that the entropy of an *n*-bit quantization of a continuous random variable is approximately

(9) $$H\left(M^{\delta_{M}}\right)\approx H\left(M\right)-\ln\left(\delta_{M}\right),$$

where $M^{\delta_{M}}$ stands for discretized random variable. As a consequence, the earthquake’s entropy, by using Equation (7) in Equation (8) and solving Equation (9), is

(10) $$H\left(M^{\delta_{M}}\right)\approx 1+\ln\left(\frac{\bar{M}-M_{c}^{u}}{\delta_{M}}\right).$$

We observe that entropy *H* depends on the difference between the average magnitude $\bar{M}$ and the completeness magnitude $M_{c}^{u}$, as reported in diverse investigations [14,15,16,18]. Since entropy must be non-negative (H≥0), requiring the argument of the logarithm in Equation (10) to be at least its minimum admissible value for $\delta_{M}=0.1$ implies

(11) $$\bar{M}-M_{c}^{u}\geq \frac{\delta_{M}}{e}\approx 0.036,$$

which is a reasonable value in the context of our analysis. Moreover, Equation (5) indicates that the *b*-exponent of the Gutenberg–Richter must satisfy

(12) b≤11.8,

which is a limit value that has not been reported in the extant literature [29,30,31,32]. Frohlich and Davis [29] analyzed four global earthquake catalogs and showed that, although the estimated *b*-values span approximately the interval 0.5≤b≤2, most tectonic seismicity is characterized by values close to unity. In volcanic environments, considerably larger *b*-values have been reported, occasionally reaching b≈3 [30]. Roberts et al. [31] pointed out that although elevated *b*-values are commonly observed in volcanic settings, incomplete earthquake catalogs or inaccurate estimates of the magnitude of completeness may artificially inflate their estimated values. Previous studies have investigated the entropy of earthquake magnitudes without incorporating the discretization correction introduced in Equation (9). Despite this omission, the qualitative behavior of the reported entropy is consistent with the results presented here [14,15]. For example, in [14], authors report that $H=-\log_{10}\left(\frac{\log_{10}\left(e\right)}{\bar{M}-M_{c}^{u}}\right)+\log_{10}\left(e\cdot \log_{10}\left(e\right)\right)$, which implies that $\bar{M}-M_{c}^{u}\geq e\cdot \log_{10}^{2}\left(e\right)\approx 0.51$ and $b\leq {\left(e\cdot \log_{10}e\right)}^{-1}\approx 0.84$, which is a very limited restriction for the *b*-parameter.

### 2.3. Rényi Entropy of Earthquake Magnitudes

In the context of information theory, a variety of extensions and generalizations of the concept of entropy have been proposed [6,28,33,34]. According to the fractal dimension analysis, Rényi’s entropy is regarded as the foundation of the concept of generalized dimensions [35]. In order to explore the behavior of this generalized entropy (and take advantage of its connection to Shannon’s entropy), it is necessary to construct the discretized version of this entropy. The Rényi entropy [7,34] for a continuous random variable *X* is defined as follows:

(13) $$H_{\alpha }\left(X\right)=\frac{1}{1-\alpha }\ln\int_{x}f^{\alpha }\left(x\right)dx,\;\alpha >0\;\&\;\alpha \neq 1,$$

with *f* a probability density function. The order parameter α controls how much the metric penalizes or rewards different parts of the distribution. That is, values close to zero assume uniformity in the probabilities, while large values tend to penalize unlikely events. If we now consider the random variable representing the magnitudes of the earthquakes to be *M* instead of *X*, substitute Equation (7) into Equation (13), and consider the correction given by Equation (9), we obtain

(14) $$H_{\alpha }=\frac{1}{1-\alpha }\ln\left\{\frac{{\left(\frac{\bar{M}-M_{c}^{u}}{\delta_{M}}\right)}^{1-\alpha }}{\alpha }\right\},\;\alpha >0\;\&\;\alpha \neq 1.$$

The case α=1 is not directly defined by this expression; however, taking the limit α→1, $H_{\alpha }$ reduces to the Shannon entropy [7], which in the present case is given by Equation (10). Therefore, throughout the remainder of this work, α=1 is considered using Equation (10). According to the discrete expression of Rényi entropy given in Equation (14), it is possible to determine the degree of uncertainty or dispersion of seismic activity within a defined time window. The order parameter, α, controls the level of irregularity or uncertainty by weighing the differences between $\bar{M}$ and $M_{c}^{u}$. Uncertainty decreases as α>1 increases. It is important to note the relationship between Rényi entropy and fractal dimensions, which stems from the fact that generalized (or Rényi) fractal dimensions are defined directly based on the scaling behavior of Rényi entropy as the resolution (window size) becomes smaller. As is widely recognized in the field of multifractal analysis, this relationship constitutes the fundamental definition: the spectrum of fractal dimensions $D_{\alpha }$ is obtained from the scaling of the Rényi entropy $H_{\alpha }$ of a partition with the window size. However, in this paper, the focus will be on a defined scale.

## 3. Data: Seismic Regions Associated with the M8.2 (Tehuantepec, 2017) and the M7.7 (Michoacan, 2022) Events

We consider the last two earthquakes with magnitudes M≥7.7. In chronological order, the first event occurred in the Gulf of Tehuantepec on 7 September 2017, with magnitude M=8.2 (see Figure 1) and a depth of 45.9 km. The studied region comprises the longitude interval [95.7W,91W] and the latitude interval [13N,16N], with an approximate area of A ≈ 169,320 km^2^. The Gulf of Tehuantepec segment of the Middle America Trench had long been recognized as a seismic gap before the M8.2 earthquake, meaning it had not produced a great event in the historic record despite being part of a very active subduction zone. Previous large earthquakes in the region included the September 1902 event and other significant shocks in 1903, 1931, and 1999, but none approached the size of the 2017 rupture. The 2017 earthquake therefore stands as the largest instrumentally recorded event in the Gulf of Tehuantepec area, breaking a long interval without large earthquakes and confirming the gap’s capacity to generate very large, damaging seismic events. Concerning the second mainshock, the left panel of the map (see Figure 1) shows the studied region for the M=7.7 earthquake that occurred in Michoacán on 19 September 2022 at a depth of 12.1 km. This region comprises the longitude interval [105.6W,102W] and the latitude interval [17N,20.5N], with an approximate area of A ≈ 147,914 km^2^. The M7.7 earthquake occurred along the same subduction segment that produced the devastating 19 September 1985 M8.1 Michoacán earthquake and its M7.5 aftershock two days later. This portion of the Middle America Trench, where the Cocos Plate subducts beneath the North American Plate, has a well-documented history of large megathrust events, including the 1985 mainshock that caused catastrophic damage in Mexico City, as well as other significant earthquakes such as the 1995 M8.0 Colima–Jalisco event to the northwest and several M≥7 shocks throughout the 20th century. The 2022 rupture, located near Coalcomán (Michoacán state) at a shallow depth, reactivated part of this historically active plate interface, underscoring the persistent seismic hazard along the Michoacán–Guerrero margin. It is noteworthy that seismic activity in the M7.7 region is predominantly attributable to the subduction of the Rivera Plate, while seismic activity in the M8.2 region is primarily attributed to the subduction of the Cocos Plate. In this study, all depth values available in the seismic catalogue were considered. The details of the depth distributions and the corresponding latitude–depth and longitude–depth relationships for each region can be found online in the Supplementary Materials at https://doi.org/10.6084/m9.figshare.33946042 (accessed on 1 October 2026).

Figure 2 presents the time series of earthquake magnitudes for the regions delineated in Figure 1, which are the focus of this study. As discussed in Section 2, the identification of the completeness magnitude is required. For this purpose, we consider the Gutenberg–Richter law in the original form expressed in Equation (1). However, in practice, two linear regimes can be identified, separated by the completeness magnitude $M_{c}$: $M\in [M_{min},M_{c}]$ and $M\in [M_{c},M_{max}]$. The latter represents the magnitude range over which the seismic catalog is considered complete. Here, $M_{min}$ and $M_{max}$ denote the minimum and maximum magnitudes reported in the catalog, respectively. In order to determine the value of $M_{c}$, we vary a threshold value $M_{X}$ over the entire magnitude range and calculate the absolute residuals from the least-squares fit for the left interval $\left[M_{min},M_{X}\right]$ and right interval $\left[M_{X},M_{max}\right]$. Subsequently, we compute the sum of these residual arrays, $S_{R}\left(M_{X}\right)$, as a function of $M_{X}$. Finally, the completeness magnitude is determined as the value of $M_{X}^{*}=M_{c}$ that minimizes $S_{R}\left(M_{X}\right)$. In Figure 3, we show a representative example of this procedure for a two-year time period for each region shown in Figure 1. Since we are interested in studying the entropy as a function of time, we applied a sliding-window method with a window size of *N* events to the regions associated with the M7.7 and M8.2 earthquakes. Consecutive windows were shifted by *k*-events. We present the results for *N* = 2000 and k=15 events resulting in an overlap of 99.25% between consecutive windows. A more exhaustive exploration of both window sizes and overlap percentage can be found in the Supplementary Materials at https://doi.org/10.6084/m9.figshare.33946042 (accessed on 1 October 2026). Our results show that the observed behavior of the Rényi entropy presents only minor changes with respect to the choice of window size and overlap. We identified the completeness magnitudes as $M_{c}=3.5$ and $M_{c}=3.8$ for the M7.7 and M8.2 regions, respectively. After applying the completeness-magnitude criterion, the resulting datasets contain 21,428 seismic events for the M8.2 region over the period 2015–2020, of which 38% occurred before and 62% after the mainshock, and 14,303 seismic events for the M7.7 region over the period 2020–2025, of which 60% occurred before and 40% after the mainshock, respectively. The difference in the total number of events is consistent with the pronounced spatial variability of seismicity along the Mexican Pacific subduction zone, with significantly lower seismicity near the Rivera–Cocos plate boundary than in the southern portion of the Mexican Pacific region [36,37,38].

## 4. Evolution of the Rényi Entropy of Earthquakes Magnitudes

### 4.1. Results for the 7 September 2017, M8.2 Earthquake

As shown in Figure 4, the calculated magnitudes $H_{\alpha }$ and $\Delta H_{1}$ (subscript stands for α=1) are plotted as a function of time for the earthquakes that occurred during the specified period (1 January 2015 to 1 January 2020), which includes the occurrence of the M8.2 earthquake. The cases of $H_{\alpha }$ for α=1, 1.5 and 2 are illustrated in Figure 4a. In the three cases under observation, entropy was found to decrease and then plateau, before abruptly increasing upon the occurrence of the M6.9 earthquake on 14 June 2017. Subsequent to this occurrence, the entropy values exhibit a moderate increase until, once more, a more pronounced abrupt increase is observed upon the occurrence of the M8.2 earthquake. Following the most recent abrupt increase, entropy undergoes a rapid growth until reaching a maximum value, after which it declines until it attains values that are lower than those recorded prior to the initial abrupt change. We also note that, as expected, $H_{\alpha }$ decreases as its order increases ($H_{1}>H_{1.5}>H_{2}$). This is because higher orders in α penalize rare events, which reduces uncertainty.

To more clearly identify changes in the increase or decrease in entropy and remove linear trends, the first entropy increment $\Delta H_{1}\left(i\right)=H_{1}\left(i+1\right)-H_{1}\left(i\right)$ for the case α=1 is calculated, where *i* denotes the window number, that is, $\Delta H_{1}$ represents the difference between the values of consecutive windows. As illustrated in Figure 4b, the evolution of the increments reveals that negative values denote a decrease, a phenomenon evident during the period preceding M8.2. However, for the inter-period between the two earthquakes, the trend shows a positive shift before the second earthquake, indicative of a fluctuating growth. It is noteworthy that, in the immediate aftermath of the M8.2 earthquake, the entropy increments undergo an abrupt decline to negative values, subsequently stabilizing around zero. A more detailed exploration of the entropy behavior as a function of different values for both window sizes and overlap percentages can be found in the Supplementary Materials at https://doi.org/10.6084/m9.figshare.33946042 (accessed on 1 October 2026). The results obtained show that the temporal evolution of Rényi entropy remains qualitatively similar when different depth intervals are considered, and a similar phenomenon occurs when the window size and the percentage overlap are varied.

In order to examine the role of earthquake depths in Rényi entropy, the distributions of said depths were analyzed (see Section S1 in the Supplementary Materials at https://doi.org/10.6084/m9.figshare.33946042 (accessed on 1 October 2026)). It was observed that the distribution of earthquake depths is bimodal, with a minimum between the peaks that separates the shallow and deep earthquakes. The shallower-depth distribution contains approximately 77% of the earthquakes in the M8.2 dataset and accounts for the main characteristics observed in the Rényi entropy results presented in this subsection. Earthquakes occurring at greater depths do not significantly affect these results.

Next, in order to illustrate the differences in the decay of the Rényi entropy as a function of the parameter α, Figure 4c presents a graphical representation of the behavior of $H_{\alpha }$ and relative entropy $H_{\alpha }^{*}=H_{\alpha }/H_{max}$ versus α for three distinct stages: before, shortly after, and after the occurrence of the mainshock. Here, $H_{max}$ denotes $H_{\alpha }$ evaluated at α=0.1. While entropy is known to decrease with increasing α, it has also been observed that the values for the three cases gradually diverge from one another. This phenomenon is evident when comparing relative entropy, which decreases more rapidly in the pre- and post-event phases compared to the during-event phase. This result suggests that uncertainty decreases more rapidly during these intervals (before and after the event), which is linked to a reduction in the differences between $\bar{M}$ and $M_{c}^{u}$. Moreover, the abrupt change in entropy observed on 19 September 2017 reveals characteristics associated with critical transitions (see Figure 4d). By considering $t_{0}$ as the time of occurrence of the M8.2 earthquake, the results indicate that the increase in $H_{\alpha }$ relative to its value at $t_{0}$, $H_{\alpha }\left(t\right)-H_{\alpha }\left(t_{0}\right)$, approximately follows the scaling law $H_{\alpha }\left(t\right)-H_{\alpha }\left(t_{0}\right)∼{\left(t-t_{0}\right)}^{c}$, where *t* denotes time (in days) and *c* is the scaling exponent. It is important to note that the scaling is independent of the value of α. The linear regression fit leads to the exponent c=0.47. It is notable that the scaling result for the entropy increments is strikingly similar to that reported by Lifshitz and Slyozov [39] (and, independently, by Wagner [40]) regarding a similar scaling for the growth time of the characteristic size of the minority-phase droplets following the phase transition.

### 4.2. Results for the 19 September 2022, M7.7 Earthquake

In this case, the period from 1 January 2020 to 1 January 2025 was analyzed, which includes the M7.7 earthquake, as illustrated in Figure 5a. In contrast to the previous case examined, this seismic event was not preceded or followed by another significant earthquake. As illustrated in Figure 5a, the evolution of Rényi entropy is depicted for three cases of the exponent α. In all three cases, it has been observed that following the M7.7 event, the entropy undergoes an abrupt increase, followed by a sustained rise until it reaches a maximum value and then decreases. Furthermore, the evolution of the first differences, as demonstrated in Figure 5b, manifestly evinces that prior to the M7.7 occurrence, the entropy differences oscillate around the neutral value, abruptly escalate post-earthquake, and then decline to negative values prior to attaining stability and oscillating around zero. The Supplementary Materials at https://doi.org/10.6084/m9.figshare.33946042 (accessed on 1 October 2026) also explores the behavior of entropy for different window sizes and overlap percentages for this event. The results also confirm that the temporal evolution of Rényi entropy remains qualitatively similar when different depth intervals are considered, and a similar phenomenon occurs when the window size and the percentage overlap are varied.

With respect to the seismic depth of earthquakes, the distribution of depths for the M7.7 earthquake is characterized by an unimodal curve, which differs from the distribution of depths for the M8.2 earthquake discussed above. The effect of these seismic events on entropy is predominantly attributed to shallow-depth events (see for details Section S1 in the Supplementary Materials at https://doi.org/10.6084/m9.figshare.33946042, accessed on 1 October 2026).

As demonstrated in the case of the M8.2, both the relative entropy $H_{\alpha }^{*}$ vs. α exhibit differentiated decays when the pre-event, during, and post-event intervals are compared. That is to say, the pre-event and post-event intervals reach a lower value of uncertainty compared to the interval during the event. Furthermore, as was the case for M8.2, the sudden change in entropy that occurred on 19 September 2022 also reveals characteristics associated with critical transitions (see Figure 5d). In this case, the increase in the Rényi entropy, $H_{\alpha }\left(t\right)-H_{\alpha }\left(t_{0}\right)$, approximately follows a scaling law of the form $H_{\alpha }\left(t\right)-H_{\alpha }\left(t_{0}\right)∼{\left(t-t_{0}\right)}^{c}$, with the same behavior for the three values of α. The linear fit yielded an exponent of c=0.29. It is noteworthy that the value of the exponent c∼1/3 is closely aligned with that observed in critical transitions [39,40].

## 5. Discussion and Conclusions

An analysis of entropy behavior during intervals in which significant-magnitude earthquakes occur has been presented. In the context of seismic activity, we have shown that Rényi entropy can be estimated in terms of the parameter *b* (the exponent of the GR law) and the control parameter α. This estimation can be made more specifically as a function of the threshold magnitude $M_{c}^{u}$, the average magnitude $\bar{M}$, and the magnitude resolution $\delta_{M}$. The results of the study indicate that, for the two earthquakes under analysis, entropy undergoes an abrupt increase once the event has occurred. Entropy decreases before the event and declines after the abrupt increase without a defined baseline. The behavior of entropy is weighted by the control parameter, α, which differentiates entropies as its value increases. This is advantageous compared to using only Shannon entropy, where events are not weighted. This distinct approach to weighting events offers insight into the behavior of entropy through the distribution of magnitudes. This behavior is rooted in the difference between $\bar{M}$ and $M_{c}^{u}$. The approach places greater emphasis on less frequent events (large magnitudes) when α is small or on more frequent events (small magnitudes) when α is larger. To assess whether the observed behavior of the Rényi entropy depends on the original temporal ordering of the seismic magnitudes, we analyzed the same time windows after randomly shuffling the magnitude series from the two examined regions. Our results show that the entropy, for each order parameter, remains approximately constant over the analyzed periods, with no abrupt changes. More details about this analysis are offered in the online Supplementary Materials at https://doi.org/10.6084/m9.figshare.33946042 (accessed on 1 October 2026).

The findings of this study are consistent with those of previous reports that utilized entropy analysis to examine seismic sequences from various locations. For instance, Varotsos et al. [9] and Sarlis et al. [10] have described how, for major earthquakes in Japan and Mexico, the entropy associated with natural time increases abruptly. Similarly, Telesca et al. [11], De Santis et al. [12] and Posadas et al. [13] have reported transitions in Shannon entropy following the occurrence of a major earthquake in the regions of Italy and Chile, respectively.

In the context of earthquake–magnitude dynamics, it is imperative to explore the relationship between Rényi and the non-extensive Tsallis entropy. The two quantities are proxies for the uncertainty and complexity of the probability distribution p(M) of observed magnitudes. An initial exploration of the behavior of Tsallis entropy (see Section S3 in the Supplementary Materials at https://doi.org/10.6084/m9.figshare.33946042 (accessed on 1 October 2026)) was conducted, resulting in the observation that both entropies manifest qualitatively similar patterns for the analyzed cases. Therefore, both measures accentuate the distinction between $\bar{M}$ and $M_{c}^{u}$ in the distribution, contingent on *q* or α. In this regard, Tsallis entropy is often useful for capturing long-range interactions in seismicity. Further research is necessary to explore this topic in greater depth. As has been documented, the fragment–asperity interaction model for the earthquake dynamics proposed in [41], in conjunction with the non-extensive entropy analysis [42,43], has demonstrated an apparent universal value of the non-extensive parameter within the range q≈ 1.2–1.6 [3,43]. It is our intention to undertake a more thorough examination of the connection between the two entropies in the near future.

Despite the existence of prior studies that have identified abrupt changes in quantities such as entropy and extent for various regions of the world, there is a necessity for research that specifically addresses this issue in specific regions, such as the Mexican Pacific coast. In this regard, our work addresses this knowledge gap by confirming that large earthquakes can be characterized by abrupt transitions in quantities such as entropy.

From a general perspective, our analysis of the transition to a major earthquake using thermodynamic and information theory measures—while considering the behavior of the system of geological faults and their interactions as an open, dissipative system—reaffirms the existence of phases such as stress accumulation, seismic quiescence, and instability, followed by the transition.

A limitation of our study is the incompleteness of the seismic catalog, given the pivotal role that the threshold magnitude plays in the entropy definition utilized in this study. Subsequent efforts are necessary to resolve the issue of expanding the catalog using a strategy involving synthetic data. In addition, the window size used in the present analysis was selected empirically. A systematic analysis of the dependence of the results on the choice of window size was beyond the scope of the present study and will be addressed in future work, together with the use of synthetic data to further assess the robustness of the results. A remarkable a is that an incomplete seismic catalog, particularly due to the under-detection of low-magnitude events, can lead to an underestimation of the *b*-value [44]. Consequently, as the completeness magnitude $M_{c}$ decreases, more low-magnitude events are included in the catalog, resulting in a lower average magnitude $\bar{M}$ and, consequently, a higher estimate of the *b*-value, as shown in Equation (5). Another implication is that both Shannon and Rényi entropies attain smaller values for catalogs with lower completeness magnitudes $M_{c}$. This behavior is consistent with the results reported in [14] where the Chilean seismic catalog has a completeness magnitude of $M_{c}=2.2$, and the reported Shannon entropy values range from approximately 0.15 to 0.45.

Using the generalized version of Shannon’s classical entropy and constructing the corresponding discrete version made it possible to analyze the behavior of complex seismic data associated with two major events. Our empirical results confirmed that changes in entropy can be identified and may be linked to microstructural changes in tectonic plates. This approach contributes to the enhancement of the relationship between information theory and seismology by offering a more applicable and scalable methodology for interpreting the complex, nonlinear precursor signals that trigger critical shifts in the Earth’s crust dynamics.

## Figures and Tables

**Figure 1:**
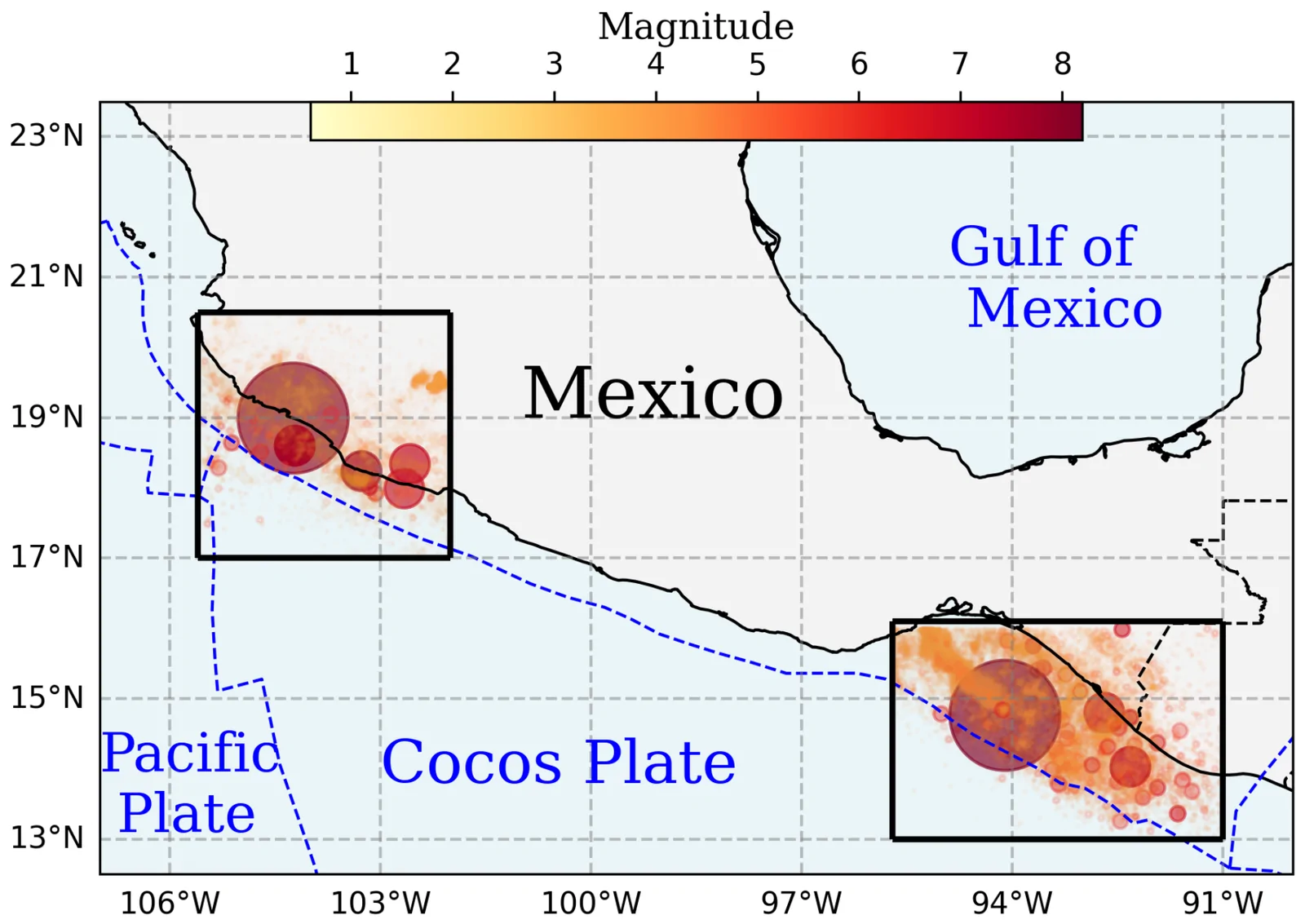
Seismicity of the two regions associated with the M7.7 Michoacán 2022 (left square), and the M8.2 Tehuantepec 2017 (right square) events from 1 January 1990 until 6 December 2024. The left-square region is delimited by the longitude interval [105.6W,102W] and the latitude interval [17N,20.5N]. The right square region is delimitated by the longitude interval [95.7W,91W] and the latitude interval [13N,16N]. The centers of the circles correspond to the epicenters of each seismic event, and the size and color of the circles correspond to the magnitude. The blue dashed indicate the boundaries of the tectonic plates.

**Figure 2:**
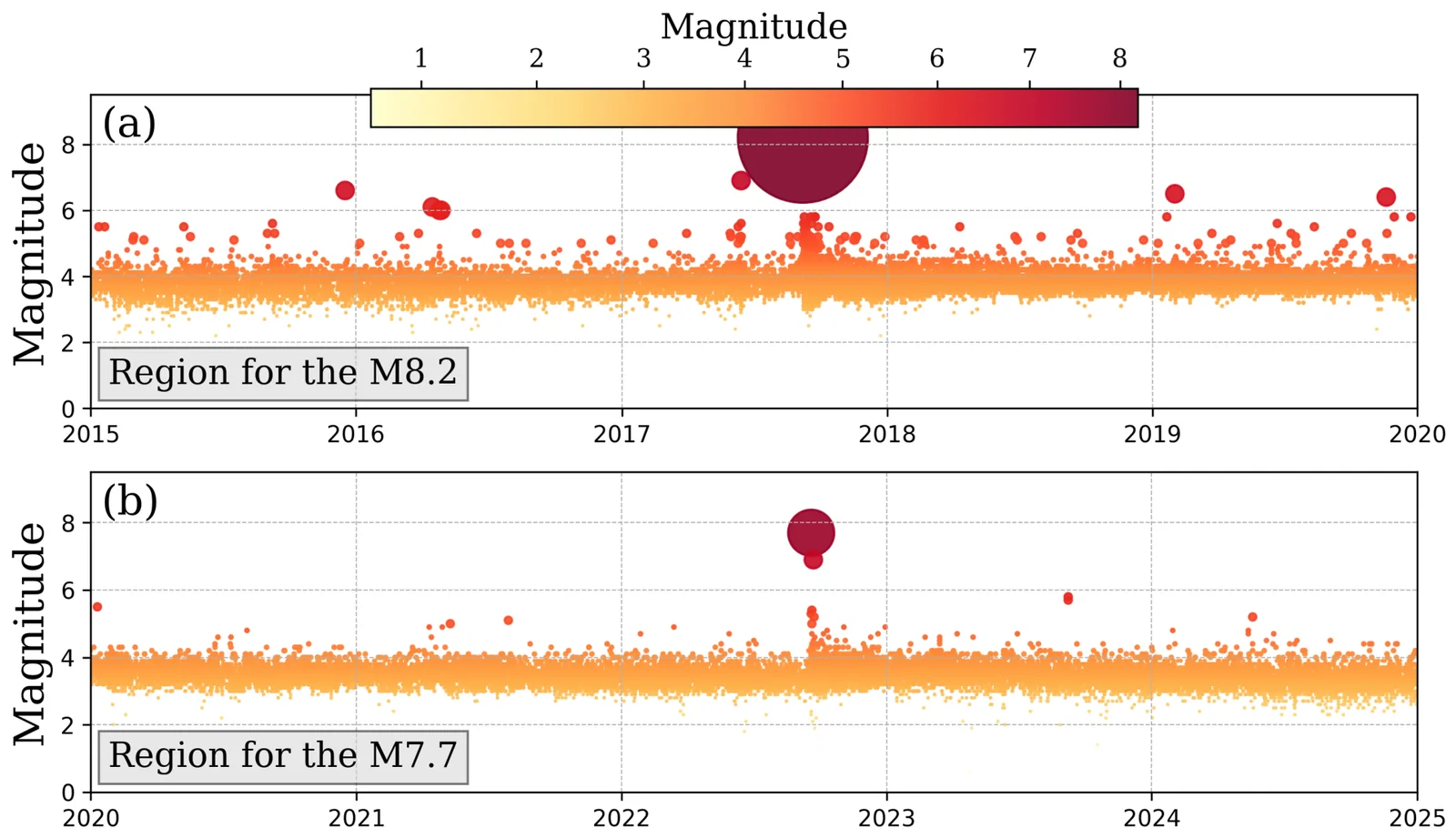
Time series of the earthquake magnitudes of the (**a**) region of associated with M8.2; (**b**) region associated with M7.7 (colors and sizes of the circles correspond to their magnitudes as shown in Figure 1).

**Figure 3:**
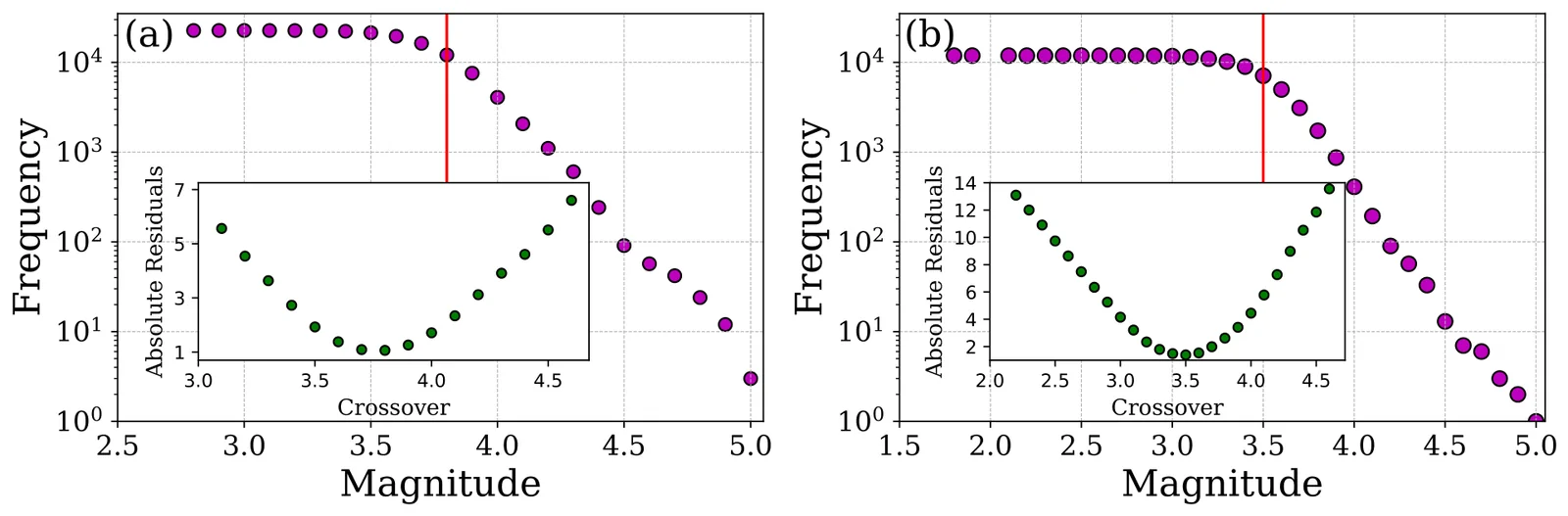
Determination of the completeness magnitude $M_{c}$. (**a**) Gutenberg–Richter distribution (purple circles) for the seismic catalog associated with the M8.2 earthquake during the period from 1 January 2018 to 1 January 2020. (Inset) Results of the residuals of the fits (green circles) as a function of the crossover magnitude. The vertical red line marks the completeness magnitude, defined as the crossover magnitude that minimizes the residuals. (**b**) As in (**a**), but for the seismic catalog associated with the M7.7 earthquake during the period from 1 June 2021 to 1 June 2023.

**Figure 4:**
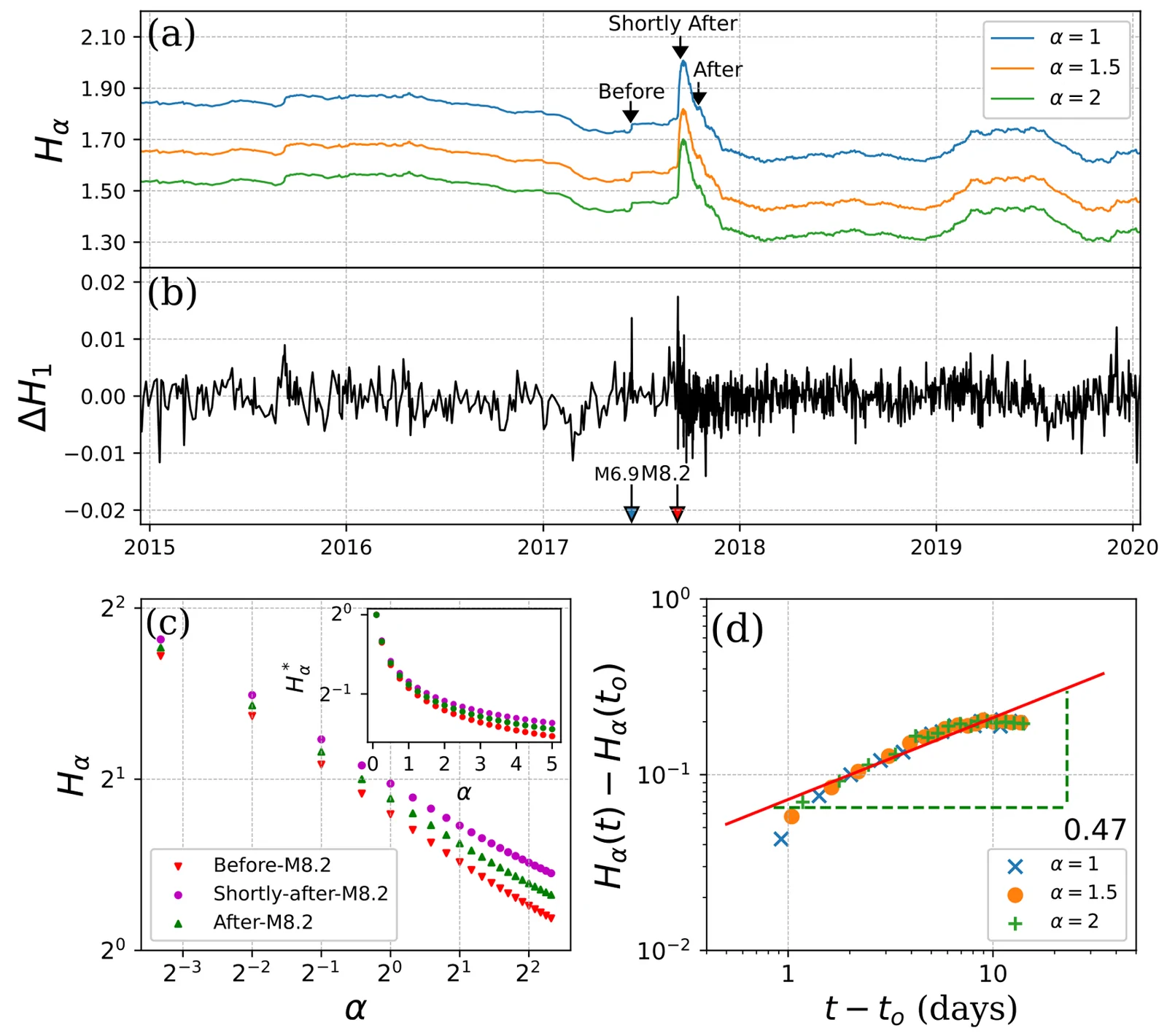
Results for the Rényi seismic entropy associated with the M8.2 earthquake. (**a**) Temporal evolution of the Rényi entropy $H_{\alpha }$ for α=1,1.5,2. (**b**) First differences in the Rényi entropy $\Delta H_{1}$. A similar behavior is observed for α=1.5 and α=2. The blue and red arrows indicate the occurrence dates of the M6.9 and M8.2 earthquakes, respectively. (**c**) The $\log_{2}-\log_{2}$ plot of $H_{\alpha }$ vs. α illustrates the rapid decay of the Rényi entropy as the parameter α increases during the “Before” stage (red downward-pointing triangles), the “Shortly after” stage (blue dots), and the “After” stage (green upward-pointing triangles). As the parameter α increases, we observe that the three cases slightly separate each other. (Inset) Relative Rényi entropy $H_{\alpha }^{*}=H_{\alpha }/H_{max}$ (with $H_{max}=H_{0.1}$) vs. α. When comparing the three cases in the main panel, it can be seen that the “Before” case decays the fastest, followed by the “After” case and then the “Shortly after” case. (**d**) Differences in $H_{\alpha }$ with respect to the entropy level $H_{\alpha }\left(t_{0}\right)$ at the time of the M8.2 mainshock. It is evident that the three cases illustrated converge to form a single curve, thereby suggesting that this scaling behavior is independent of the parameter α. The solid red line represents the least-squares fit, with a slope of 0.47 and a coefficient of determination of $R^{2}=0.91$. The completeness magnitude used in the analysis is $M_{c}=3.8$.

**Figure 5:**
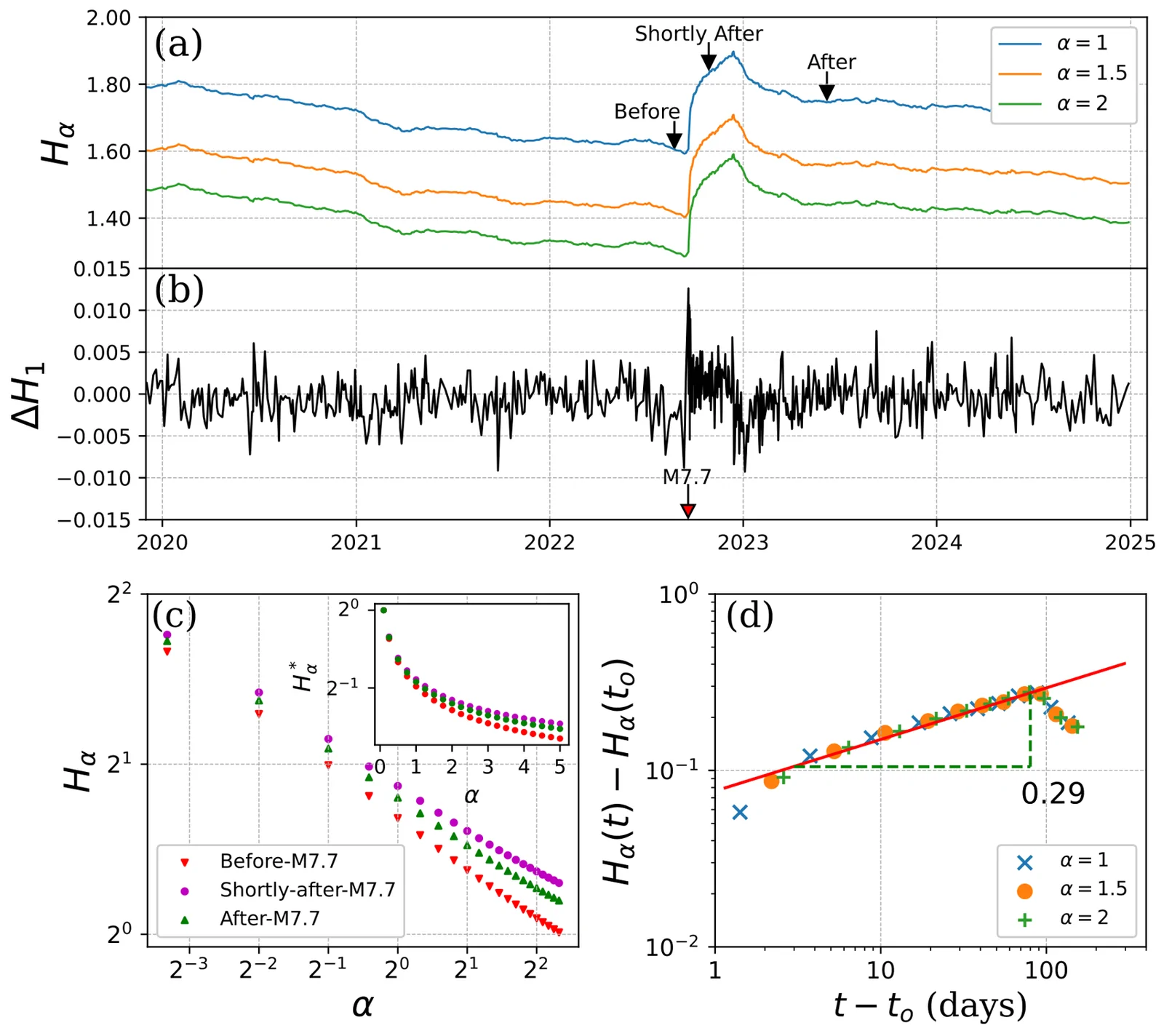
Results for the Rényi seismic entropy associated with the M7.7 earthquake. (**a**) Temporal evolution of the Rényi entropy $H_{\alpha }$ for α=1,1.5,2. (**b**) First differences in the Rényi entropy for $H_{1}$. A similar behavior is observed for α=1.5 and α=2. The red arrow indicates the occurrence date of the M7.7 earthquake. (**c**) The $\log_{2}-\log_{2}$ plot of $H_{\alpha }$ vs. α illustrates the rapid decay of the Rényi entropy as the parameter α increases during the “Before” stage (red downward-pointing triangles), the “Shortly after” stage (blue dots), and the “After” stage (green upward-pointing triangles). As the parameter α increases, we observe that the three cases slightly separate each other. (Inset) Relative Rényi entropy $H_{\alpha }^{*}=H_{\alpha }/H_{max}$ (with $H_{max}=H_{0.1}$) vs. α. When comparing the three cases in the main panel, it can be seen that the “Before” case decays the fastest, followed by the “After” case and then the “Shortly after” case. (**d**) Differences in the Rényi entropy with respect to the entropy level $H_{\alpha }\left(t_{0}\right)$ at the time of the M7.7 mainshock for the three values of α. The solid red line represents the least-squares fit, with a slope of 0.29 and a coefficient of determination of $R^{2}=0.96$. The completeness magnitude used in the analysis is $M_{c}=3.5$.

## Data Availability

The data used in this study are available from the official website of the Servicio Sismológico Nacional de la Universidad Nacional Autónoma de México at http://www.ssn.unam.mx (accessed on 16 January 2025).

## Supplementary Materials

The following supporting information can be downloaded at: https://doi.org/10.6084/m9.figshare.33946042 (accessed on 1 October 2026) [45].

## Abbreviations

The following abbreviations are used in this manuscript:

M8.2	Mainshock of seismic magnitude M = 8.2
M7.7	Mainshock of seismic magnitude M = 7.7
*M*	Earthquake magnitude
*a*	Gutenberg–Richter *a*-value
*b*	Gutenberg–Richter *b*-value
GR law	Gutenberg–Richter law
$log_{10}$	Logarithmic function with base 10
ln	Natural logarithmic function
$M_{c}$	Completeness magnitude
N(M)	Cumulative number of earthquakes with magnitude greater or equal to *M*
S(M)	Survivor function of earthquake magnitude
F(M)	Cumulative distribution function of earthquake magnitude
P(M)	Probability density function of earthquake magnitude
$\bar{M}$	Average earthquake magnitude
$M_{c}^{u}$	Utsu-corrected completeness magnitude
$\delta_{M}$	Magnitude resolution
M	Continuous earthquake magnitude random variable
H(M)	Shannon entropy of the continuous earthquake magnitude random variable
$M^{\delta_{M}}$	Discretized earthquake magnitude random variable
$H(M^{\delta_{M}})$	Shannon entropy of the discretized earthquake magnitude random variable
*e*	Euler’s number, the base of the natural exponential function
$H_{\alpha }$	Rényi entropy of order α of the discretized earthquake magnitude random variable
α	Rényi entropy order parameter
$M_{min}$	Minimum magnitude reported in the seismic catalog
$M_{max}$	Maximum magnitude reported in the seismic catalogue
$M_{X}$	Threshold magnitude value
$S_{R}\left(M_{X}\right)$	Sum of the residual arrays as a function of $M_{X}$
$M_{X}^{*}$	Threshold magnitude value for which $M_{X}=M_{c}$
$\Delta H_{1}$	Increase in Shannon entropy for α=1
τ	Number of lag windows used for $\Delta H_{1}$
$H_{max}$	Maximum value of $H_{\alpha }$ for a given α
$t_{0}$	Occurrence time of the M8.2 or M7.7 earthquake
$H^{*}$	Relative entropy $H_{\alpha }/H_{max}$

## References

[1] Stein S., Liu M., Calais E., Li Q. (2009). Mid-continent earthquakes as a complex system. Seismol. Res. Lett..

[2] Abe S., Suzuki N. (2006). Complex-network description of seismicity. Nonlinear Processes Geophys..

[3] Chelidze T., Vallianatos F., Telesca L. (2018). Complexity of Seismic Time Series: Measurement and Application.

[4] Perez-Oregon J., Angulo-Brown F., Sarlis N.V. (2020). Nowcasting avalanches as earthquakes and the predictability of strong avalanches in the Olami-Feder-Christensen model. Entropy.

[5] Turcotte D.L., Malamud B.D., Lee W.H.K., Kanamori H., Jennings P.C., Kisslinger C. (2002). Earthquakes as a Complex System. International Handbook of Earthquake and Engineering Seismology, Part A.

[6] Shannon C.E. (1948). A mathematical theory of communication. Bell Syst. Tech. J..

[7] Rényi A. (1961). On Measures of Entropy and Information. Proceedings of the 4th Berkeley Symposium on Mathematical Statistics and Probability, Volume 1: Contributions to the Theory of Statistics.

[8] Tsallis C. (1988). Possible generalization of Boltzmann-Gibbs statistics. J. Stat. Phys..

[9] Varotsos P.A., Sarlis N.V., Nagao T. (2024). Complexity measure in natural time analysis identifying the accumulation of stresses before major earthquakes. Sci. Rep..

[10] Sarlis N.V., Skordas E.S., Varotsos P.A., Ramírez-Rojas A., Flores-Márquez E.L. (2018). Natural time analysis: On the deadly Mexico M8. 2 earthquake on 7 September 2017. Phys. A Stat. Mech. Appl..

[11] Telesca L., Lapenna V., Lovallo M. (2004). Information entropy analysis of seismicity of Umbria-Marche region (Central Italy). Nat. Hazards Earth Syst. Sci..

[12] De Santis A., Cianchini G., Favali P., Beranzoli L., Boschi E. (2011). The Gutenberg–Richter law and entropy of earthquakes: Two case studies in Central Italy. Bull. Seismol. Soc. Am..

[13] Posadas A., Morales J., Posadas-Garzon A. (2021). Earthquakes and Entropy: Characterization of Occurrence of Earthquakes in Southern Spain and Alboran Sea. Chaos Interdiscip. J. Nonlinear Sci..

[14] Posadas A., Pasten D., Vogel E.E., Saravia G. (2023). Earthquake hazard characterization by using entropy: Application to northern Chilean earthquakes. Nat. Hazards Earth Syst. Sci..

[15] Feng L.H., Luo G.Y. (2009). The relationship between seismic frequency and magnitude as based on the Maximum Entropy Principle. Soft Comput..

[16] De Santis A., Abbattista C., Alfonsi L., Amoruso L., Campuzano S.A., Carbone M., Cesaroni C., Cianchini G., De Franceschi G., De Santis A. (2019). Geosystemics view of earthquakes. Entropy.

[17] Gutenberg B., Richter C.F. (1944). Frequency of earthquakes in California. Bull. Seismol. Soc. Am..

[18] Aki K. (1965). Maximum likelihood estimate of b in the formula log N = a-bM and its confidence limits. Bull. Earthq. Res. Inst. Univ. Tokyo.

[19] Nava F., Ávila-Barrientos L., Márquez-Ramírez V., Torres I., Zúñiga F. (2018). Sampling uncertainties and source b likelihood for the Gutenberg-Richter b value from the Aki-Utsu method. J. Seismol..

[20] Perez-Oregon J., Muñoz-Diosdado A., Rudolf-Navarro A., Guzmán-Sáenz A., Angulo-Brown F. (2018). On the possible correlation between the Gutenberg-Richter parameters of the frequency-magnitude relationship. J. Seismol..

[21] Mitsui Y. (2024). Stable estimation of the Gutenberg–Richter b-values by the b-positive method: A case study of aftershock zones for magnitude-7 class earthquakes. Earth Planets Space.

[22] Taroni M. (2023). Estimating the magnitude of completeness of earthquake catalogs using a simple random variable transformation. Seism. Rec..

[23] Tinti S., Gasperini P. (2024). The estimation of b-value of the frequency–magnitude distribution and of its 1 *σ* intervals from binned magnitude data. Geophys. J. Int..

[24] van der Elst N.J. (2021). B-positive: A robust estimator of aftershock magnitude distribution in transiently incomplete catalogs. J. Geophys. Res. Solid Earth.

[25] Bender B. (1983). Maximum likelihood estimation of b values for magnitude grouped data. Bull. Seismol. Soc. Am..

[26] Utsu T. (1965). A method for determining the value of b in a formula log n = a = bM showing the magnitude frequency relation for earthquakes. Geophys. Bull. Hokkaido Univ..

[27] Breiman L. (1992). Probability.

[28] Cover T.M., Thomas J.A. (2006). Elements of Information Theory.

[29] Frohlich C., Davis S.D. (1993). Teleseismic b values; or, much ado about 1.0. J. Geophys. Res. Solid Earth.

[30] McNutt S.R. (2005). Volcanic seismology. Annu. Rev. Earth Planet. Sci..

[31] Roberts N.S., Bell A.F., Main I.G. (2015). Are volcanic seismic b-values high, and if so when?. J. Volcanol. Geotherm. Res..

[32] Glazner A.F., McNutt S.R. (2021). Relationship between dike injection and b-value for volcanic earthquake swarms. J. Geophys. Res. Solid Earth.

[33] Amigó J.M., Balogh S.G., Hernández S. (2018). A brief review of generalized entropies. Entropy.

[34] Tabass M.S., Borzadaran G.M., Amini M. (2016). Renyi entropy in continuous case is not the limit of discrete case. Math. Sci. Appl. E-Notes.

[35] Feder J. (2013). Fractals.

[36] Mota-Palomino R., Andrieux J., Bonnin J. (1986). Bosquejo sismotectónico del sur de México. Geofís. Int..

[37] Eissler H.K., McNally K.C. (1984). Seismicity and tectonics of the Rivera plate and implications for the 1932 Jalisco, Mexico, earthquake. J. Geophys. Res. Solid Earth.

[38] Flores-Márquez E.L., Ramírez-Rojas A., Sigalotti L.D.G. (2024). Non-Extensive Statistical Analysis of Seismicity on the West Coastline of Mexico. Fractal Fract..

[39] Lifshitz I.M., Slyozov V.V. (1961). The kinetics of precipitation from supersaturated solid solutions. J. Phys. Chem. Solids.

[40] Wagner C. (1961). Theorie der alterung von niederschlägen durch umlösen (Ostwald-reifung). Z. Elektroch. Ber. Bunsenges. Phys. Chem..

[41] Sotolongo-Costa O., Posadas A. (2004). Fragment-asperity interaction model for earthquakes. Phys. Rev. Lett..

[42] Posadas A., Sotolongo-Costa O. (2023). Non-extensive entropy and fragment–asperity interaction model for earthquakes. Commun. Nonlinear Sci. Numer. Simul..

[43] Pasten D., Vogel E.E., Saravia G., Posadas A., Sotolongo O. (2023). Tsallis entropy and mutability to characterize seismic sequences: The case of 2007–2014 northern Chile earthquakes. Entropy.

[44] Lombardi A. (2021). A normalized distance test for co-determining the completeness magnitude and b-value of earthquake catalogs. J. Geophys. Res. Solid Earth.

[45] Mariz A.M. (1992). On the irreversible nature of the tsallis and renyi entropies. Phys. Lett. A.

